# Is Cumulative Load Associated with Injuries in Youth Team Sport? A Systematic Review

**DOI:** 10.1186/s40798-022-00516-w

**Published:** 2022-09-16

**Authors:** Katie Sniffen, Kemba Noel-London, Melody Schaeffer, Oluwatoyosi Owoeye

**Affiliations:** 1 Department of Physical Therapy and Athletic Training, Saint Louis University, St. Louis, MO USA; 2 Advanced Health Data Institute, Saint Louis University, St. Louis, MO USA; 3 Department of Health Management and Policy, Saint Louis University, St. Louis, MO USA

**Keywords:** Youth team sport, Load management, Injury prevention

## Abstract

**Background:**

High cumulative external and internal load may predispose athletes to increased risk for injury across a variety of sports, competition levels, and age groups. However, evidence of an association between cumulative load and injury in youth sport remains inconclusive. The objective of this study was to determine the current evidence for cumulative load and injury risk relationships in youth team sport through a systematic review of the existing literature.

**Methods:**

A systematic review of the literature was performed following the Preferred Reporting Items for Systematic Reviews and Meta-Analysis (PRISMA) guidelines. Literature searches were conducted in PubMed, Web of Science, SCOPUS, and CINAHL for relevant articles published between January 2010 and April 2021. The authors conducted independent review and quality assessment of the eligible studies. Eleven articles evaluating youth (less than 18 years old) team sport were included for qualitative synthesis.

**Results:**

Fifty-nine percent (*n* = 39/66) of the relationships assessed revealed an association between cumulative load and injury across the team sports studied, including the presence of load–injury associations in 84% (*n* = 16/19) of assessments in youth soccer. Of those relationships where an association was present, 79% (*n* = 31/39) were positive associations between cumulative load and injury. Risk of bias assessment scores ranged from three to six out of seven possible (median = 5) for cohort studies and from four to seven out of 10 possible (median = 5.5) for cross-sectional studies.

**Conclusions:**

There is some evidence for a positive association between load and injury in youth team sport. Youth soccer was the most studied team sport, and a substantial number of positive load–injury associations were reported. Current evidence lacks consistency in the measures and metrics used in defining load–injury relationships.

*Trial Registration* PRISMA ID - CRD42020203622.

**Supplementary Information:**

The online version contains supplementary material available at 10.1186/s40798-022-00516-w.

**Key Points**

- There is some evidence that a positive association between cumulative workload and injury exists in youth team sport.
- Mitigating overload (excessive accumulation of load) in youth team sport may be a successful injury prevention strategy.
- Current evidence lacks consistency in the measures and metrics used in defining load–injury relationships.

## Background

Injury prevention is crucial to maximize the benefits of sports participation among youth and to protect them from dire future health consequences [1, 2]. Athletic load monitoring and management is a target for injury prevention and performance optimization in team sports [3, 4]. Although studies evaluating the relationships between load and injury have become more prevalent in recent years, research evidence to inform best practices for injury prevention remains limited; especially in competitive youth sport [5–7].

Load is any modifiable exertional exposure (relating to training, practice and/or competition) imposed on an athlete to elicit improvements in performance measures (i.e., speed, endurance), and it can be experienced in the form of external or internal load [8]. External load metrics seek to quantify the amount of work completed by the athlete through measurements such as total distance covered, accelerations, or non-locomotor actions (i.e., number of jumps). Internal load, a psycho-physiological response to external stressors, is commonly examined through athlete-reported rating of perceived exertion (RPE). This metric is typically calculated as the product of the athlete’s RPE for a training session and the duration of the training session expressed in “arbitrary units” or “exertional units” [8, 9].

Load can be calculated as cumulative load [also known as accumulated or absolute load (i.e., total load per week)] or relative load [i.e., the acute-chronic workload ratio (ACWR)], and both calculations have been used to evaluate load–injury relationships [5, 10]. However, recently, the ACWR has been suggested to have statistical limitations that make it less of a valid choice in conducting load management research and practice [11–13]. Accordingly, this systematic review focused on studies that evaluated the load–injury relationship using cumulative load calculations.

Conceptual models and evolving evidence relating to load–injury relationships show that high cumulative external and internal load, albeit good for performance, may predispose athletes to increased risk for injury across a variety of sports, competition levels, and age groups [4, 14–16]. Much of the work done evaluating the load–injury relationship for elite, adult athletes suggests positive associations whereby as load increases, injury incidence increases [17]. When preseason training loads are decreased from one season to the next, injury rates reduce substantially while maintaining or improving measures of performance [18]. Changes in training load of greater than 15% from week to week result in 21–49% increases in injury risk [19]. The linear load–injury relationship has evolved to consider a quadratic relationship where certain thresholds of training load have a protective effect on injury risk. Subsequently, relatively low and excessively high training loads can both lead to increases in injury incidence [19].

Despite a growing body of literature, comprehensive evidence of an association between cumulative load and injury in youth sport remains elusive and reviews to date are limited to soccer [20]. There is currently no systematic review evaluating the relationship between cumulative load and injury risk in youth team sport. Thirty eight percent of children aged 6–12 years old participate in sport each year with sport-related injuries occurring at a rate of 76.7 episodes per 1000 persons in a population of a similar age group (5–15 years old) [21, 22]. High-school aged athletes experience 2.29 time-loss injuries per 1000 athlete exposures [23]. Physical, physiological, and psychological maturation occurring during adolescence may affect the response to training load compared to that of elite, adult athletes. This population also uniquely participates in sport. Young people aged 6–12 years old and 12–17 years old participate in an average of 1.79 and 1.96 sports per year, respectively [22]. Over 36% of high school athletes highly specialize in sport with more than 60 competitions per year [24]. The tendency toward year-long training patterns, crowded competition calendars, and multi-team/sport participation among youth team sport athletes may uniquely reduce this population’s potential for recovery and subsequently increase risk of injury.

The objective of this study was to determine the current evidence for cumulative load and injury risk relationships in youth team sport through a systematic review of the existing literature. Specifically, this systematic review reports on the population of youth team sport athletes, workload independent variables, and injury outcomes.

## Methods

### Searches

A systematic review of the literature was performed following the Preferred Reporting Items for Systematic Reviews and Meta-Analysis (PRISMA) guidelines (ID: CRD42020203622, Additional file 2: Table S1). Literature searches were conducted in PubMed using medical subject headings (MESH) and in Web of Science, SCOPUS, and CINAHL using topic, keyword, and subject searches, respectively. The following search terms were included in Boolean search strategies:

“(Adolescen* OR youth OR young OR child) AND (athlet*) AND (rugby OR soccer OR football OR volleyball OR handball OR basketball OR “team sport”*) AND (train*) AND (load OR intens* OR volume OR duration OR workload OR rep OR exertion) AND (injur* OR risk*)”

The full search strategy is included in Additional file 1.

### Study Inclusion and Exclusion Criteria

Studies were included if they were original research articles with an observational study design and published in English in peer-reviewed journals from January 2010 to April 2021. The studied sample must have included team sport athletes at any level less than 18 years old. Team sport was operationally defined as sport in which a group of more than two athletes compete against another group of athletes. Independent variables must have included measures of either internal or external load (i.e., number of sessions completed, distance covered during session, jump count, rate of perceived exertion, duration) and calculated as absolute or cumulative loads. Studies calculating only relative load such as the ACWR were excluded due to recent evidence refuting the validity of such measures [12, 25]. Dependent variable measurements must have included a measure of injury occurrence (i.e., number of injuries) or pain, and measures of association with the independent variable (i.e., relative risk, odds ratio). Injury classifications (i.e., contact vs non-contact; sudden-onset vs gradual-onset) were not specified to accommodate for the diversity of injury definitions used in injury surveillance. Outcomes measures of illness were excluded.

Each article was independently reviewed for inclusion by two of the authors in two phases: (1) title and abstract review, and (2) full-text review. All authors participated in the independent review of at least half the articles. Discrepancies between authors’ decisions were resolved via a third author’s review. The reference list of articles included after full-text review was searched independently by two authors for any potential eligible studies that were not identified via the electronic literature search.

### Study Quality Assessment

A quality assessment of each study’s risk of bias was conducted independently by two authors using adapted versions of the Newcastle Ottawa Scale (NOS) for case–control and cohort studies, as appropriate [26]. The NOS for case–control studies utilizes a star rating system to indicate study quality among components of selection, comparability, and exposure. This assessment tool was adapted by the authors and used for cross-sectional studies. The NOS for cohort studies follows the same star rating system among components of cohort selection, cohort comparability, and outcome assessment. A maximum of nine stars can be awarded across eight items, with a higher number of stars indicating higher methodological quality. As with other systematic reviews of load and injury relationships, the NOS for cohort studies was modified to reflect the nature of load exposure [20, 27]. The original Item Two, related to the “selection of the non-exposed cohort,” was removed since each study’s target population was exposed to a team sport, thus removing the possibility of a non-exposed cohort. The original Item Five, related to “comparability of cohorts on the basis of the design or analysis,” was also removed due to its relation to Item 2. For the original Item 3, related to “ascertainment of exposure,” written self-report was awarded a star for studies using internal load measures since subjective measurement [i.e., session rate of perceived exertion (sRPE)] is the accepted data collection procedure for internal load. A novel item was added to each scale to assess inclusion of injury definition and a star was awarded if the study included a definition of injury. In total, seven stars were possible across seven items in the modified NOS for cohort studies, and 10 stars were possible across nine items in the modified NOS for cross-sectional studies. Each evaluation criterion with descriptions can be found in Additional file 3: Table S2 and Additional file 4: Table S3. Any discrepancies in reviewer assessment were resolved by consensus.

### Data Extraction

Data extraction was completed by the lead author (KS) and reviewed by the senior author (OO). The following information was extracted from each study: study details (author, year, study design, duration of follow-up); population characteristics (age, sex, sport); load definition (internal and/or external); injury/pain definition (time loss, non-time loss); and measure of association (relative risk, odds ratio).

### Data Synthesis and Presentation

Data extracted from each study were synthesized to describe the cumulative number of reported relationships between cumulative load and injury. Each studied relationship between cumulative load and injury was categorized as either reporting the presence of an association or reporting no presence of association. When the presence of an association was present, it was further categorized as either a positive, negative, or bidirectional association. Counts of each type of association were accumulated to summarize the nature of the relationship between cumulative load and injury. Due to the diversity of load measures and injury definitions, meta-analysis was not pursued.

## Results

**Fig. 1:**
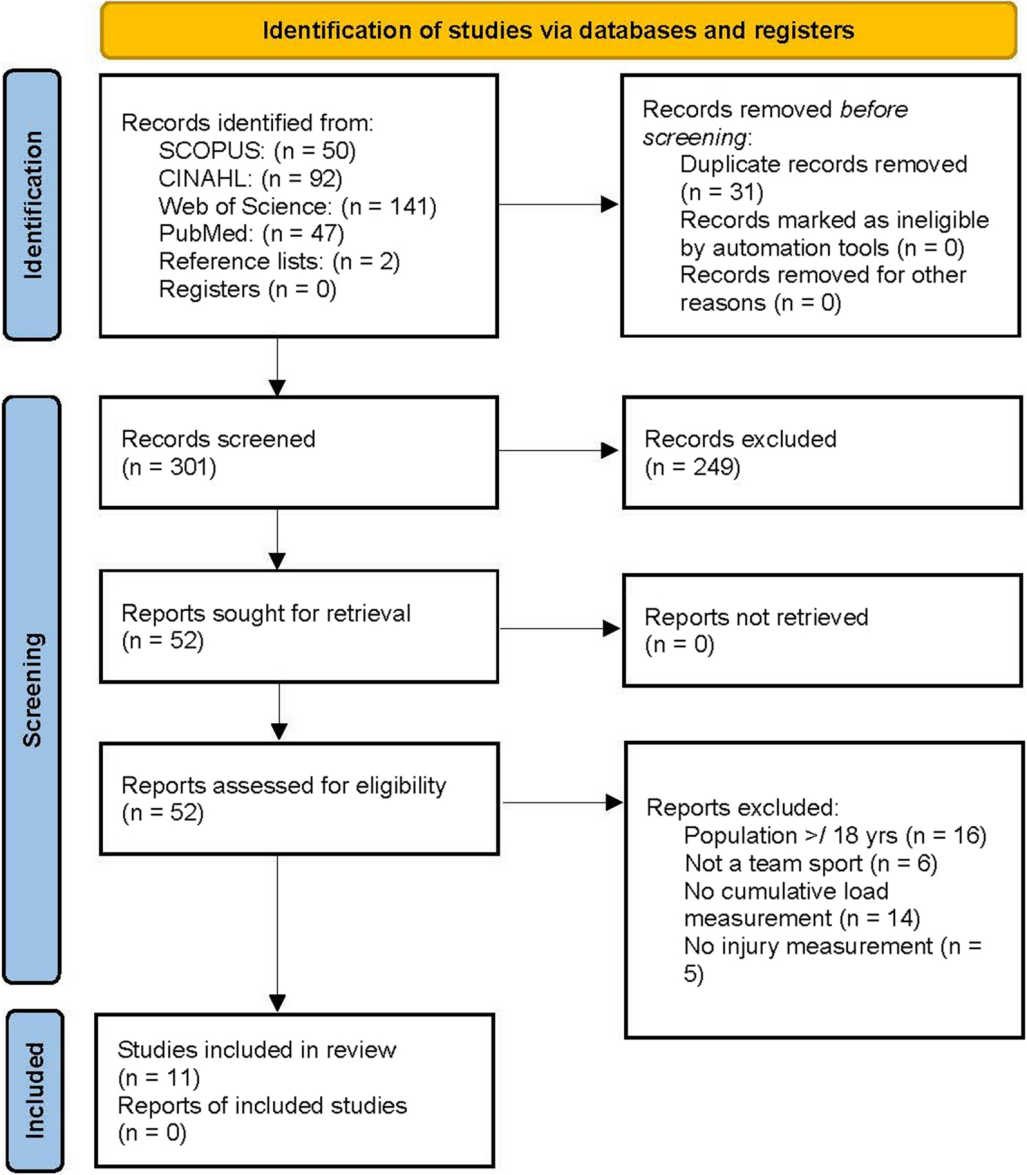
PRISMA flow diagram

The literature search was conducted between September 2020 and April 2021 and returned 330 articles across the four databases. Thirty-one articles were identified as duplicates, resulting in 299 titles and abstracts screened for inclusion. After 248 articles were excluded, the full text of 51 articles was assessed for eligibility. Two articles were later identified from reference lists during full text review. Of the two additional abstracts reviewed, one article was excluded, resulting in a total of 52 articles for full text review. Forty-one articles were excluded with a final 11 studies remaining for qualitative synthesis. The flow diagram of the inclusion process and reasons for full text exclusion can be found in Fig. 1, [28].

The majority of included studies were prospective cohort studies (*n* = 9/11, 82%). Soccer was the most studied team sport (*n* = 4/11, 36%) and males were the most commonly studied population (*n* = 9/11, 82%). External (*n* = 6/11, 55%) and internal load (*n* = 5/11, 45%) measures were nearly equally assessed. About half of studies restricted their injury definition to time-loss injuries only. Others allowed for any complaint that required medical attention, including pain. Risk of bias assessment scores ranged from three to six out of seven possible (median = 5) for cohort studies and from three to seven out of 10 possible (median = 5.5) for cross-sectional studies (Additional file 5: Table S4). Results from the data extraction are presented in Tables 1 and 2.

**Table 1:** Description of included studies

Reference	NOS	Study design	Sport	Population*	Time period	Load definition		Injury definition
						Internal	External	
Ahmun et al. [29]	6	Prospective cohort	Cricket	39 males (17.5 [0.80] yrs)	3 years, 5 tours	Session RPE: 10-point RPE × session duration (min) Acute workload: 3 day avg Chronic workload: 14 day avg		Medical attention conditions
Bowen et al. [10]	4	Prospective cohort	Football (Soccer)	32 males (17.3 [0.9] yrs)	2 seasons		Total distance High-speed distance Total load Accelerations	Time-loss injuries
Brink et al. [30]	4	Prospective cohort	Soccer	58 males (15–18 yrs)	2 seasons	Session RPE: 15-point RPE × session duration (hr) Weekly load: sum of sessions over 1 week		Any physical complaint from soccer, regardless of need for medical attention or time-loss
Frisch et al. [31]	4	Cross-sectional	Volleyball	175 female high school athletes	4 years		Volume of contacts: self-reported number of serves or spikes in practices during a typical week	Volleyball-related shoulder pain: traumatic and non-traumatic
Hartwig et al. [32]	3	Prospective cohort	Rugby	93 males (14–16 yrs)	1 year		Training volume (min/wk) Match volume (min/wk) Weekly volume (min/wk)	Rugby-related, time-loss injury or pain
Lathlean et al. [5]	5	Prospective cohort	Australian football	290 males (17.7 [0.3] yrs	1 year	Session load: 10-point RPE × session duration (m) 1-wk load 2-wk load 3-wk load 4-wk load		Time-loss injuries
O’Keeffe et al. [7]	6	Prospective cohort	Gaelic football	97 males (13.4 [1.1] yrs)	1 season	Load: modified RPE × session duration (m) 1-wk load 2-wk load 3-wk load 4-wk load		Any time-loss injury sustained during training or competition
Orr and Cheng [33]	5	Prospective cohort	Rugby	122 males baseline cohort: n = 368 (15.8 [1.0])	1 season		Weekly physical activity (time spent in team training, additional sporting endeavors, exercise or work/manual labor)	Any physical or medical condition that occurs during participation in rugby league match or training activities that requires medical treatment or results in a missed match or training participation
Ristolainen et al. [6]	7	Cross-sectional	Multi-sport^a^	549 males (14–16 yrs) 528 females (14–16 yrs)	1 year		Training hours per week during training season Training hours per week during competition season Amount of competitions	Acute time-loss injuries; overuse injuries
Visnes and Barr [34]	5	Prospective cohort	Volleyball	69 males; 72 females (16.8 [0.8] yrs)	4 years		Number of hours of volleyball, beach volleyball, strength, jump training, and other training Number of sets played in matches during the last week	Jumper's knee
Watson et al. [35]	5	Prospective cohort	Soccer	75 females (15.5 ± 1.6 yrs)	20 weeks	Fatigue Session RPE (duration min × intensity 1–10) Daily, weekly (7 day) rolling avg, monthly (28 day) rolling avg		Time-loss injuries

*NOS* Newcastle Ottawa scale, *RPE* Rate of perceived exertion

*Age reported as available in source article (i.e., mean [SD], or range)

^a^Top team sports (> 12% participation) included soccer, ice-hockey, floorball

**Table 2:** Data extraction from included studies

Reference	Statistic	95% Confidence interval	Statistic adjusted for covariates	Presence of association	Direction
Ahmun et al. [29]	Acute WL: RR = 2.51 Chronic WL: RR = 1.48	Acute WL: (1.70,3.70) Chronic WL: (1.01, 2.70)	Acute WL: No Chronic WL: No	Acute WL: Yes Chronic WL: Yes	Acute WL: Positive Chronic WL: Positive
Bowen et al. [10]	*Overall injuries*: High TD 4-wk: RR = 1.64 Low TD 1-wk: RR = 0.25 Mod-High 4-wk HSD: RR = 2.14 Mod-High 1-wk HSD: RR = 1.73 Low HSD 1-wk: RR = 0.30 High TL 1-wk: RR = 1.65 Low TL 1-wk: RR = 0.27 Very High ACC 3-wk: RR = 3.84 Low ACC 3-wk: RR = 0.31	*Overall injuries*: High TD 4-weekly: (1.05, 2.58) Low TD 1-weekly (0.11, 0.82) Mod-High HSD: (1.31, 3.50) Mod-High HSD 1-weekly: (1.06, 2.84) Low HSD 1-wk: (0.13, 0.68) High TL 1-wk: (1.04, 2.62) Low TL 1-wk: (0.12, 0.60) Very High ACC 3-wk: (1.57, 9.41) Low ACC 3-wk: (0.13, 0.76)	*Overall injuries*: High TD 4-wk: No Low TD 1-wk: No Mod-High HSD 4-wk: No Mod-High HSD 1-wk: No Low HSD 1-wk: No High TL 1-wk: No Low TL 1-wk: No Very High ACC 3-wk: No Low ACC 3-wk: No	*Overall injuries*: High TD 4-wk: Yes Low TD 1-wk: Yes Mod-High HSD 4-wk: Yes Mod-High HSD 1-wk: Yes Low HSD 1-wk: Yes High TL 1-wk: Yes Low TL 1-wk: Yes Very High ACC 3-wk: Yes Low ACC 3-wk: Yes	*Overall injuries*: High TD 4-wk: Positive Low TD 1-wk: Negative Mod-High HSD 4-wk: Positive Mod-High HSD 1-wk: Positive Low HSD 1-wk: Negative High TL 1-wk: Positive Low TL 1-wk: Negative Very High ACC 3-wk: Positive Low ACC 3-wk: Negative
Brink et al. [30]	*Traumatic injury*: Duration: RR = 1.14 Load: RR = 1.01 *Overuse injury*: Duration: RR = 1.07 Load: RR = 1.01	*Traumatic injury*: Duration: (1.06, 1.23) Load: (1.00, 1.02) *Overuse injury*: Duration: (0.98, 1.18) Load: (1.00, 1.01)	*Traumatic injury*: Duration: No Load: No *Overuse injury*: Duration: No Load: No	*Traumatic injury*: Duration: Yes Load: Yes *Overuse injury*: Duration: No Load: No	*Traumatic injury*: Duration: Positive Load: Positive *Overuse injury*: Duration: NA Load: NA
Frisch et al. [31]	Unadjusted: OR = 1.3 Adjusted for years playing and out of season weight lifting: OR = 1.3	Unadjusted: (1.1–1.7) Adjusted: (0.9–1.7)	Unadjusted: No Adjusted: Yes	Unadjusted: Yes Adjusted: No	Adjusted: Positive Adjusted: NA
Hartwig et al. [32]	Training volume: OR = 1.03 Match volume: OR = 1.41 Weekly volume: OR = 1.19	Training volume: (0.78–1.33) Match volume: (1.14–1.74) Weekly volume: (0.93–1.51)	Training volume: Yes Match volume: Yes Weekly volume: Yes	Training volume: No Match volume: Yes Weekly volume: No	Training volume: NA Match volume: Positive Weekly volume: NA
Lathlean et al. [5]	Session load linear: OR = 0.64 Session load quadratic: OR = 1.44 1-wk load linear: OR = 0.56 1-wk load quadratic: OR = 1.43 2-wk load: OR = 1.01 3-wk load: OR = 0.99 4-wk load: OR = 0.92	Session load linear: (0.49–0.83) Session load quadratic: (1.11–1.88) 1-wk load linear: (0.42–0.73) 1-wk load quadratic: (1.07–1.92) 2-wk load: 0.914–1.11) 3-wk load: (0.89–1.11) 4-wk load: (0.83–1.03)	Session load linear: No Session load quadratic: No 1-wk load linear: No 1-wk load quadratic: No 2-wk load: No 3-wk load: No 4-wk load: No	Session load linear: Yes Session load quadratic: Yes 1-wk load linear: Yes 1-wk load quadratic: Yes 2-wk load: No 3-wk load: No 4-wk load: No	Session load linear: Negative Session load quadratic: Bidirectional 1-wk load linear: Negative 1-wk load quadratic: Bidirectional 2-wk load: NA 3-wk load: NA 4-wk load: NA
O'Keeffe et al. [7]	1-wk load: OR = 2.75 2-wk load: OR = 2.57 3-wk load: OR = 2.57 4-wk load: OR = 2.57	1-wk load: (1.00–7.59) 2-wk load: (0.94–7.07) 3-wk load: (0.94–7.07) 4-wk load: (0.94–7.07)	1-wk load: Yes 2-wk load: Yes 3-wk load: Yes 4-wk load: Yes	1-wk load: Yes 2-wk load: No 3-wk load: No 4-wk load: No	1-wk load: Positive 2-wk load: NA 3-wk load: NA 4-wk load: NA
Orr and Cheng [33]	Weekly PA did not correlate with number of match injuries (*r* = − 0.03, *p* = 0.83) Weekly PA did not correlate with match injuries per match hour (*r* = − 0.07, *p* = 0.60)		Number of match injuries: No Number of match injuries per hour: No	Number of match injuries: No Number of match injuries per hour: No	Number of match injuries: NA Number of match injuries per hour: NA
Ristolainen et al. [6]	*Acute injuries*				
	*Boys*: *Training season hrs/wk*: 3–6 h/wk: ref 7–14 h/wk: OR = 1.57 15 + hr/wk: OR = 2.16 *Competition season hrs/wk*: 3–6 h/wk: ref 7–14 h/wk: OR = 1.39 15 + hr/wk: OR = 1.67 *Competitions*: 7–19: ref 20–39: OR = 1.48 40 + : OR = 2.02	*Boys*: *Training season hrs/wk*: 3–6 h/wk: ref 7–14 h/wk: (1.06–2.32) 15 + hr/wk: (1.28–3.64) *Competition season hrs/wk*: 3–6 h/wk: ref 7–14 h/wk: (0.94–2.04) 15 + hr/wk: (0.99–2.81) *Competitions*: 7–19: ref 20–39: (0.89–2.47) 40 + : (1.21–3.37)	*Boys*: *Training season hrs/wk*: 3–6 h/wk: No 7–14 h/wk: No 15 + hr/wk: No *Competition season hrs/wk*: 3–6 h/wk: No 7–14 h/wk: No 15 + hr/wk: No *Competitions*: 7–19: No 20–39: No 40 + : No	*Boys*: *Training season hrs/wk*: 3–6 h/wk: NA 7–14 h/wk: Yes 15 + hr/wk: Yes *Competition season hrs/wk*: 3–6 h/wk: NA 7–14 h/wk: No 15 + hr/wk: No *Competitions*: 7–19: NA 20–39: No 40 + : Yes	*Boys*: *Training season hrs/wk*: 3–6 h/wk: NA 7–14 h/wk: Positive 15 + hr/wk: Positive *Competition season hrs/wk*: 3–6 h/wk: NA 7–14 h/wk: NA 15 + hr/wk: NA *Competitions*: 7–19: NA 20–39: NA 40 + : Positive
Ristolainen [6]	*Overuse injuries*				
	*Boys*: *Training season hrs/wk*: 3–6 h/wk: ref 7–14 h/wk: OR = 1.91 15 + hr/wk: OR = 2.14 *Competition season hrs/wk*: 3–6 h/wk: ref 7–14 h/wk: OR = 1.64 15 + hr/wk: OR = 2.26 *Competitions*: 7–19: ref 20–39: OR = 1.21 40 + : OR = 1.42	*Boys*: *Training season hrs/wk*: 3–6 h/wk: ref 7–14 h/wk: (1.24–2.94) 15 + hr/wk: (1.24–3.71) *Competition season hrs/wk*: 3–6 h/wk: ref 7–14 h/wk: (1.08–2.49) 15 + hr/wk: (1.31–3.90) *Competitions*: 7–19: ref 20–39: (0.71–2.06) 40 + : (0.8–2.41)	*Boys*: *Training season hrs/wk*: 3–6 h/wk: No 7–14 h/wk: No 15 + hr/wk: No *Competition season hrs/wk*: 3–6 h/wk: No 7–14 h/wk: No 15 + hr/wk: No *Competitions*: 7–19: No 20–39: No 40 + : No	*Boys*: *Training season hrs/wk*: 3–6 h/wk: NA 7–14 h/wk: Yes 15 + hr/wk: Yes *Competition season hrs/wk*: 3–6 h/wk: NA 7–14 h/wk: Yes 15 + hr/wk: Yes *Competitions*: 7–19: NA 20–39: No 40 + : No	*Boys*: *Training season hrs/wk*: 3–6 h/wk: NA 7–14 h/wk: Positive 15 + hr/wk: Positive *Competition season hrs/wk*: 3–6 h/wk: NA 7–14 h/wk: Positive 15 + hr/wk: Positive *Competitions*: 7–19: NA 20–39: NA 40 + : NA
Ristolainen [6]	*Acute injuries*				
	*Girls* *Training season hrs/wk*: 3–6 h/wk: ref 7–14 h/wk: OR = 1.65 15 + hr/wk: OR = 2.04 *Competition season hrs/wk*: 3–6 h/wk: ref 7–14 h/wk: OR = 1.77 15 + hr/wk: OR = 1.91 *Competitions*: 7–19: ref 20–39: OR = 1.29 40 + : OR = 0.92	*Girls* *Training season hrs/wk*: 3–6 h/wk: ref 7–14 h/wk: (1.11–2.45) 15 + hr/wk: (1.10–3.76) *Competition season hrs/wk*: 3–6 h/wk: ref 7–14 h/wk: (1.17–2.67) 15 + hr/wk: (1.05–3.48) *Competitions*: 7–19: ref 20–39: (0.77–2.15) 40 + : (0.48–1.77)	*Girls* *Training season hrs/wk*: 3–6 h/wk: No 7–14 h/wk: No 15 + hr/wk: No *Competition season hrs/wk*: 3–6 h/wk: No 7–14 h/wk: No 15 + hr/wk: No *Competitions*: 7–19: No 20–39: No 40 + : No	*Girls* *Training season hrs/wk*: 3–6 h/wk: NA 7–14 h/wk: Yes 15 + hr/wk: Yes *Competition season hrs/wk*: 3–6 h/wk: NA 7–14 h/wk: Yes 15 + hr/wk: Yes *Competitions*: 7–19: NA 20–39: No 40 + : No	*Girls* *Training season hrs/wk*: 3–6 h/wk: NA 7–14 h/wk: Positive 15 + hr/wk: Positive *Competition season hrs/wk*: 3–6 h/wk: NA 7–14 h/wk: Positive 15 + hr/wk: Positive *Competitions*: 7–19: NA 20–39: NA 40 + : NA
Ristolainen [6]	*Overuse injuries*				
	*Girls* *Training season hrs/wk*: 3–6 h/wk: ref 7–14 h/wk: OR = 1.42 15 + hr/wk: OR = 1.23 *Competition season hrs/wk*: 3–6 h/wk: ref 7–14 h/wk: OR = 1.33 15 + hr/wk: OR = 1.68 *Competitions*: 7–19: ref 20–39: OR = 1.60 40 + : OR = 1.57	*Girls* *Training season hrs/wk*: 3–6 h/wk: ref 7–14 h/wk: (0.95–2.11) 15 + hr/wk: (0.66–2.30) *Competition season hrs/wk*: 3–6 h/wk: ref 7–14 h/wk: (0.88–2.01) 15 + hr/wk: (0.92–3.05) *Competitions*: 7–19: ref 20–39: (0.95–2.71) 40 + : (0.81–3.05)	*Girls* *Training season hrs/wk*: 3–6 h/wk: No 7–14 h/wk: No 15 + hr/wk: No *Competition season hrs/wk*: 3–6 h/wk: No 7–14 h/wk: No 15 + hr/wk: No *Competitions*: 7–19: No 20–39: No 40 + : No	*Girls* *Training season hrs/wk*: 3–6 h/wk: NA 7–14 h/wk: No 15 + hr/wk: No *Competition season hrs/wk*: 3–6 h/wk: NA 7–14 h/wk: No 15 + hr/wk: No *Competitions*: 7–19: NA 20–39: No 40 + : No	*Girls* *Training season hrs/wk*: 3–6 h/wk: NA 7–14 h/wk: NA 15 + hr/wk: NA *Competition season hrs/wk*: 3–6 h/wk: NA 7–14 h/wk: NA 15 + hr/wk: NA *Competitions*: 7–19: NA 20–39: NA 40 + : NA
Visnes and Barr [34]	Total training volume: OR = 1.61 Volleyball training: OR = 1.72 Number of sets: OR = 3.88	Total training volume: (1.10–2.36) Volleyball training: (1.18–2.53) Number of sets: (1.80–8.40)	Total training volume: Yes Volleyball training: Yes Number of sets: Yes	Total training volume: Yes Volleyball training: Yes Number of sets: Yes	Total training volume: Positive Volleyball training: Positive Number of sets: Positive
Watson et al. [35]	*Training load* *Univariable* Daily: OR = 1.91 Prior day: OR = 1.42 Weekly: OR = 1.62 Monthly: OR = 1.13 *Multivariable* Daily: OR = 1.98 Prior day: OR = 1.38	*Training load* *Univariable* Daily: (1.40–2.63) Prior day: (1.04–1.95) Weekly: (1.16–2.29) Monthly: (0.75–1.67) *Multivariable* Daily: (1.42–2.78) Prior day: (1.01–1.88)	*Training load* *Univariable* Daily: No Prior day: No Weekly: No Monthly: No *Multivariable* Daily: Yes Prior day: Yes	*Training load* *Univariable* Daily: Yes Prior day: Yes Weekly: Yes Monthly: No *Multivariable* Daily: Yes Prior day: Yes	*Training load* *Univariable* Daily: Positive Prior day: Positive Weekly: Positive Monthly: NA *Multivariable* Daily: Positive Prior day: Positive

*WL* Workload, *TD* Total distance, *HSD* High speed distance, *TL* Total load, *ACC* Accelerations, *PA* Physical activity, *RR* Relative risks, *OR* Odds ratio, *NA* Not applicable, *hr/wk*: hours per week

**Fig. 2:**
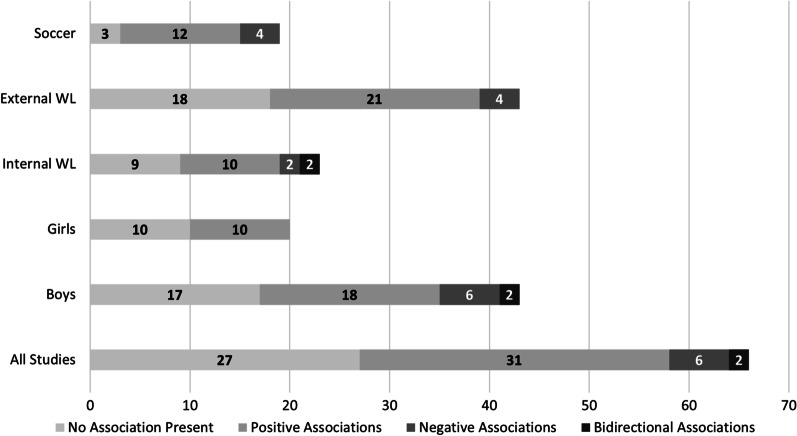
Counts of association between load and injury in youth sport.* WL* workload

Overall, 66 load–injury relationships were assessed across the 11 studies. Many of the relationships assessed revealed a statistically significant association between cumulative load and injury (*n* = 39/66, 59%); however, a considerable number of studies (*n* = 27/66, 41%) reported no association. The presence of an association held true in studies stratified by sex (boys vs. girls) and exposure (internal vs external load), as well as within soccer (Fig. 2). Of those relationships where a statistically significant association was present, 79% (*n* = 31/39) were positive associations between cumulative load and injury where increases in cumulative load were associated with increases in injury occurrence. One hundred percent (*n* = 10/10) of associations in female populations were positive relationships.

## Discussion

This systematic review synthesized the most recent and relevant literature evaluating the relationship between cumulative load and injury in youth team sport. Many of the studies evaluated suggest a statistically significant positive association between load and injury in youth team sport. This finding is consistent with the growing body of evidence suggesting an association between load and injury in sport and provides some evidence to support load management (i.e., monitoring and adjustment) as a viable injury prevention strategy in youth team sport [3, 14, 36]. Beyond the risk of gradual and sudden onset injuries, overload in youth sport may result in medical problems, including illnesses, sleep disturbance, and overtraining syndrome [37].

Some of the studies included in the current systematic review showed conflicting evidence as to the true direction of this association. There are two of reasons for this discrepancy in results. First, the causal pathway between the commonly used psycho-physiological measure of internal load in the form of sRPE and local tissue mechanical stress–strain response towards adaptation or mal-adaptation is complex [16, 38]. In particular, the degree to which load relates to injury via the mechanical load-response pathway, the psycho-physiological load-response pathway, or both is variable. Additionally, damage from tissue fatigue and trauma may influence the outcome along these pathways [38].

Second, varying and inconsistent measures and metrics used to define load and injury present challenges in defining the true relationship. Although load can be calculated in absolute/cumulative and relative terms in both internal and external load contexts, we chose the cumulative load calculation given that current recommendations for load and sports exposure are based on cumulative load [39]. However, there is still variability in how cumulative load is measured and the type of load data captured (i.e., external vs internal load). Both internal and external load metrics in these studies varied in the duration of accumulation (i.e., 1 week, 28 day, 4 week). External workload metrics were further varied by the chosen measurement unit, including distance, accelerations, and non-locomotor activities (i.e., jumps). Furthermore, despite existing consensus statements providing acceptable definitions of injury, there was wide variance in injury definitions with some studies only accounting for time-loss injuries and other taking a broader approach for all ailments requiring medical attention [40]. Any of these factors could account for the discrepancies reported across studies included in this review. Future research should address the highlighted methodological limitations in existing literature to advance knowledge on the association between load and injury risk in youth team sports.

### Implications for Practice

The implications of a potential positive association between cumulative load and injury in youth team sport, including a substantial positive association in youth soccer, have practical applications in community youth sport settings and warrant attention from coaches, parents, school administrators, and health care professionals that facilitate youth team sport participation. Effective communication and shared responsibilities among stakeholders would maximize positive health outcomes. Mitigating excessive accumulation of load in youth team sport may be a successful injury prevention strategy. Reducing the risk of injury would enable uninterrupted participation in physical activity and team sport, prevent undesirable health care costs, and avoid long-term health implications of orthopedic injury.

Although the current primary intervention for injury prevention in youth team sport is neuromuscular training warm-up exercise programs, the findings from this systematic review support load management strategies as an additional injury prevention intervention for this population [3, 41, 42]. The complexity around the etiology of sport injuries warrants that multiple interventions are considered for injury risk mitigation [43, 44]. In addition to team-based neuromuscular training warm-up exercise programs, youth sport managers, coaches, parents, and sports medicine professionals should consider pragmatic load management strategies for injury prevention in individual athletes. Simple strategies such as moderating total sessions per week and restricting or reducing participation hours could be implemented for at-risk youth athletes. It is also important to note that low training loads that do not sufficiently prepare the athlete for their performance demands may also increase the risk of injury and a more individualized approach to managing load response in youth team sport athletes may be warranted [19, 45].

While load management may be a daunting task in youth sport, it is worth the effort for improved performance, increased chances of team success, and ultimately injury prevention and health protection in youth. Although more research is needed to determine best practices for load management across youth sports, current recommended participation guidelines by DiFiori and colleagues should be considered by stakeholders for implementation to further reduce the risk of injuries in youth team sport [39].

### Strengths

To our knowledge, this is the first systematic review of the load–injury relationship across multiple youth team sports. Strengths of this study include a robust independent review process at multiple steps in data collection. Literature searches were conducted in four different databases, improving the saturation of the literature search. Utilizing the PRISMA protocol ensured transparency in the literature search and data extraction process. An all-inclusive search strategy related to study design, all team sports, and injury definitions likely resulted in more included studies and subsequently increased understanding of the load–injury relationship in youth team sport. Most studies included in this review (9/11 studies) utilized a prospective study design, which represents a high-level of evidence regarding the nature of the association between cumulative load and injury. The studies included had moderately low risk of bias based on the NOS quality assessment review.

### Limitations

This systematic review is not without limitations; some are already highlighted, but specific limitations are discussed further. We were unable to conduct a meta-analysis due to the diversity of measures, metrics, and varied definitions of injury and load used across studies. Because the literature search was limited to English language, a publication and language bias is possible. While use of the NOS risk-of-bias assessment was used, the inclusion of a variety of study designs and modification of the tool limited the ability to truly compare all studies or have complete confidence in the tool’s validity. The studies included in this review limit the ability of the conclusions to represent all youth team sports, diverse races and ethnicities, and various cultural norms related to youth sport participation. Disparities across youth team sport support services (i.e., sports medicine staff) may misrepresent the nature of the relationship between cumulative load and injury due to inconsistencies in load management, access to appropriate health care, and subsequently, reporting load and injuries.

### Future Research

Future research should take into consideration the methodological limitations in existing literature to build upon the knowledge base of the load–injury relationship. In general, more research is needed to better understand this relationship in youth team sport. Due to the differences in duration and intensity across team sports, more studies for each of the team sports should be conducted to enable sport-specific recommendations on workload thresholds. Additionally, injury definitions should follow the proposals outlined in consensus statements to enable comparisons across studies. Consideration should be made for meaningful covariates that may play a role in injury risk. Sport specialization among youth has emerged as a concern for increased injury risk. Whether youth athletes compete in one sport or many, it is not uncommon for an athlete to participate on multiple teams over the course of a single season. Considering the prevalence of sport specialization and multi-sport participation, future research should account for load accumulated outside of the single team considered in the study.

To advance current knowledge in load–injury relationships for best practice, priority should be given to examining cumulative load thresholds in specific youth team sports using consistent or comparable injury definitions and load measures. For example, current participation guidelines and weekly hours of load thresholds for youth basketball are mostly based on expert consensus [39]. There is a need to validate these recommendations. Also, given the multifactorial nature of sport injury, a better understanding of load–injury relationships within complex models is essential [43, 44]. Future research should consider evaluating load monitoring and mediation as an integral component of a prospective multifactorial screening assessment of individual athletes to maximize injury prevention benefits.

## Conclusion

There is some evidence for a positive association between load and injury in youth team sport. Youth soccer was the most studied team sport, and a substantial number of positive load–injury associations were reported. Addressing the problem of overload in individual athletes using pragmatic load management is crucial to protect athletes’ health. The strength of existing evidence is weakened by the limitations in the studies evaluated. This systematic review highlights the need to increase both the number and the rigor of studies across youth team sport populations using consistent definitions of load and injury. Being able to make evidence-based and practical recommendations related to load thresholds should be a priority in efforts to mitigate the risk of injury and maximize performance in youth team sport. In addition to team-based neuromuscular training warm-up exercise, stakeholders should consider pragmatic load management strategies as an additional injury prevention intervention in youth team sport.

## Supplementary Information

**Additional file 1:** Search strategy.

**Additional file 2: Table S1.** PRISMA-P checklist.

**Additional file 3: Table S2.** Modified Newcastle Ottawa scale for cohort studies.

**Additional file 4: Table S3.** Modified Newcastle Ottawa scale for cross-sectional studies.

**Additional file 5: Table S4.** Modified Newcastle Ottawa scale scores.

## Data Availability

All data generated or analyzed during this study are included in this published article [and its supplementary information files].

## Abbreviations

ACWR: Acute-chronic workload ratio
PRISMA: Preferred reporting items for systematic reviews and meta-analysis
NOS: Newcastle Ottawa scale
sRPE: Session rate of perceived exertion
