# Supplementary material for: Is Cumulative Load Associated with Injuries in Youth Team Sport? A Systematic Review
Source: Sports Med Open. 2022 Sep 16;8:117. doi: 10.1186/s40798-022-00516-w (PMC9481825; doi:10.1186/s40798-022-00516-w)
Supplement: Supplementary file 1 — Additional file 1: Search strategy. [file 40798_2022_516_MOESM1_ESM.pdf]

Search Terms

Population			Independent Variable	Comparison (none)	Outcome
Youth	Team Sport	Athletes	Workload		Injury
Adolescent	Rugby		Load		Risk
Young	Soccer		Intensity		
Child	Football		Volume		
	Volleyball		Duration		
	Basketball		RPE		
	Handball		Exertion		

Search Strategy

SCOPUS

KEY((adolescen* OR youth OR young OR child) AND (athlet*) AND (rugby OR soccer OR football OR volleyball OR handball OR basketball OR "team sport"*) AND (train*) AND (load OR intens* OR volume OR duration OR workload OR rep OR exertion) AND (injur* OR risk*)) AND DOCTYPE(ar) AND PUBYEAR > 2009 AND (LIMIT-TO (LANGUAGE,"English"))

Web of Science

(TS=((adolescen* OR youth OR young OR child) AND (athlet*) AND (rugby OR soccer OR football OR volleyball OR handball OR basketball OR "team sport"*) AND (train*) AND (load OR intens* OR volume OR duration OR workload OR rep OR exertion) AND (injur* OR risk*))) AND LANGUAGE: (English) AND DOCUMENT TYPES: (Article)

Timespan: 2010-2020. Indexes: SCI-EXPANDED, SSCI, A&HCI, ESCI.

PubMed

("Adolescent"[Mesh] OR "Child"[Mesh]) AND ("Athletes"[Mesh]) AND ("Basketball"[Mesh] OR "Football"[Mesh] OR "Soccer"[Mesh] OR "Volleyball"[Mesh] OR "Youth Sports"[Mesh]) AND (load OR intens* OR volume OR duration OR workload OR rpe OR exertion) AND ("Wounds and Injuries"[Mesh]) Filters: Journal Article, English, from 2010 – 2020

CINHAL

((MH “Adolescence+”) OR (MH “Child+”)) AND (MH “Athletes+”) AND ((MH "Basketball") OR (MH "Football") OR (MH "Soccer") OR (MH "Volleyball") OR (MH "Rugby") OR (MH "Team Sports+") OR (MH "Handball")) AND ("load" OR "intens*" OR "volume" OR "duration" OR "workload" OR "rpe" OR (MH "Exertion+")) AND (MH “Wounds and Injuries+”)

Limiters - Scholarly (Peer Reviewed) Journals; Published Date: 20100101-20201231
Expanders - Apply equivalent subjects
Search modes - Boolean/Phrase
