# Supplementary material for: Is Cumulative Load Associated with Injuries in Youth Team Sport? A Systematic Review
Source: Sports Med Open. 2022 Sep 16;8:117. doi: 10.1186/s40798-022-00516-w (PMC9481825; doi:10.1186/s40798-022-00516-w)
Supplement: Supplementary file 2 — Additional file 2: Table S1. PRISMA-P checklist. [file 40798_2022_516_MOESM2_ESM.pdf]

PRISMA-P (Preferred Reporting Items for Systematic review and Meta-Analysis Protocols) 2015 checklist: recommended items to address in a systematic review protocol*

Section and topic	Item No	Checklist item	
**ADMINISTRATIVE INFORMATION**			
Title:			
Identification	1a	Identify the report as a protocol of a systematic review	How Much is Too Much in Youth Team Sport? A Systematic Review
Update	1b	If the protocol is for an update of a previous systematic review, identify as such	NA
Registration	2	If registered, provide the name of the registry (such as PROSPERO) and registration number	CRD42020203622
Authors:			
Contact	3a	Provide name, institutional affiliation, e-mail address of all protocol authors; provide physical mailing address of corresponding author	Katie Sniffen, MS, ATC; Saint Louis University; 3545 Lafayette Ave, St. Louis, MO 63104; katie.sniffen@health.slu.edu<br>Oluwatoyosi Owoeye, PhD, PT; Saint Louis University; olu.owoeye@health.slu.edu
Contributions	3b	Describe contributions of protocol authors and identify the guarantor of the review	Katie Sniffen: guarantor, protocol development, data collection, analysis, interpretation, manuscript draft and revision<br>Oluwatoyosi Owoeye: protocol development, study review, analysis, manuscript revision<br>Melody Scheaffer: study review, manuscript revision<br>Kemba Noel-London: study review, manuscript revision
Amendments	4	If the protocol represents an amendment of a previously completed or published protocol, identify as such and list changes; otherwise, state plan for documenting important protocol amendments	If amendment of the protocol is needed, we will provide the date of each amendment, a description of the changes made, and a rationale for each within this section.
Support:			
Sources	5a	Indicate sources of financial or other support for the review	NA
Sponsor	5b	Provide name for the review funder and/or sponsor	NA
Role of sponsor or funder	5c	Describe roles of funder(s), sponsor(s), and/or institution(s), if any, in developing the protocol	NA
**INTRODUCTION**			
Rationale	6	Describe the rationale for the review in the context of what is already known	Internal and external workload exceeding player tolerance or capacity is significantly associated with increased injury risk across a variety of

			sports, competition levels, and age groups. There is a lack of consensus about the threshold of workload capacity and onset of injury in youth team sports.
Objectives	7	Provide an explicit statement of the question(s) the review will address with reference to participants, interventions, comparators, and outcomes (PICO)	What are the workload thresholds that result in increased risk of injury among youth team sport athletes?<br>P: youth team sport athletes<br>I: internal and external workloads<br>C: N/A<br>O: injury: all injuries; acute injuries; overuse injuries; non-contact injuries

METHODS

Eligibility criteria	8	Specify the study characteristics (such as PICO, study design, setting, time frame) and report characteristics (such as years considered, language, publication status) to be used as criteria for eligibility for the review	Studies will be selected according to the following criteria:<br><br>**Study Design**<br>Included studies will be original research articles published in peer-review journals.<br><br>Case studies, case reports, and other reviews will be excluded.<br><br>**Population**<br>We will include studies on team sport athletes younger than 18years old.<br><br>**Intervention**<br>Studies included will have assessed internal and/or external workload measures. Internal workload measures include rate of perceived exertion (RPE) and heartrate or heartrate variability. External load measures include absolute/cumulative duration of activity (hours, days, weeks). Measures of acute:chronic workload ratios will be excluded due to recent evidence refuting the validity of such measures.<br><br>Single sports specialization will be considered as a secondary workload variable; however, studies examining sport specialization alone will be excluded.<br><br>**Comparison**

			No comparisons will be assessed in this review<br><br>**Outcome**<br>We will include studies that assess the prevalence and/or incidence of injury in relationship to the intervention.<br><br>**Setting**<br>Studies will be included if they take place in the context of youth team sport (i.e. basketball, volleyball, soccer, football, field hockey, lacrosse, handball)<br><br>**Timeframe**<br>We will limit our search to studies published between 2010-2021<br><br>**Language**<br>Our search will be limited to those articles published in English.
Information sources	9	Describe all intended information sources (such as electronic databases, contact with study authors, trial registers or other grey literature sources) with planned dates of coverage	A literature search will be conducted in PubMed using medical subject heading (MESH) and in Web of Science, SCOPUS, and CINHAL using topic, keyword, and subject searches respectively. We will review the reference list of all identified studies to ensure literature saturation.
Search strategy	10	Present draft of search strategy to be used for at least one electronic database, including planned limits, such that it could be repeated	Appropriate keywords and/or MESH terms will be identified by the research team and incorporated into a draft search query that will be reviewed by a librarian not otherwise affiliated with the research. Once finalized, this search strategy will be adapted to each database.<br><br>Draft SCOPUS Search:<br><br>(adolescen* OR youth OR young OR child) AND (athlet*) AND (rugby OR soccer OR football OR volleyball OR handball OR basketball OR “team sport*”) AND (train*) AND (load OR intens* OR volume OR duration OR workload OR rpe OR exertion) AND (injur* OR risk*)
Study records:			
Data management	11a	Describe the mechanism(s) that will be used to manage records and data throughout the review	KS will manage records of the literature search. Identified articles from the search in each database will be uploaded to EndNote X9. The library will be shared with all other authors to access and review.
Selection	11b	State the process that will be used for selecting studies (such as two	Authors will independently review titles and abstracts of identified

process		independent reviewers) through each phase of the review (that is, screening, eligibility and inclusion in meta-analysis)	studies against the established inclusion criteria. Full text copies of studies identified as relevant to the research question will be obtained for full, independent review by the authors. Any disagreement in including a study will be resolved through discussion and majority agreement. Reviewers will not be blind to study title, authors, or institution.
Data collection process	11c	Describe planned method of extracting data from reports (such as piloting forms, done independently, in duplicate), any processes for obtaining and confirming data from investigators	Included studies will undergo data extraction by one author. A second author will review data extraction. Data extraction tables will be saved in a OneDrive folder. KS will be responsible for contacting investigators for obtaining and confirming data as needed.
Data items	12	List and define all variables for which data will be sought (such as PICO items, funding sources), any pre-planned data assumptions and simplifications	Population:<br>• age (continuous, 0-17 years)<br>• sex: dichotomous (male, female)<br>• sport: categorical (basketball, football, handball, rugby, soccer, volleyball, other)<br>Intervention:<br>• Absolute/cumulative workloads: continuous (i.e. number of sessions completed, distance covered during session, jump count, rate of perceived exertion)<br>• Exclude: Acute:Chronic Workload Ratios
Outcomes and prioritization	13	List and define all outcomes for which data will be sought, including prioritization of main and additional outcomes, with rationale	• Injury:<br>o Number of injuries (continuous)<br>o Non-contact, contact (dichotomous)<br>o Acute, chronic (dichotomous)<br>o Illness excluded<br>• Injury type: (if data allows)<br>o Sprain, strain, fracture, contusion (categorical)<br>• Injured body part (if data allows)<br>o Upper extremity, lower extremity, torso, head (Categorical)<br>• Relative risks and odds ratios
Risk of bias in individual studies	14	Describe anticipated methods for assessing risk of bias of individual studies, including whether this will be done at the outcome or study level, or both; state how this information will be used in data synthesis	Two independent reviewers will use a modified version of the Newcastle Ottowa Scale (NOS) tool to assess the quality of each individual study. If a disagreement on the quality assessment is present, a third reviewer will complete the assessment, and a consensus will be determined.

			NOS Documentation
Data synthesis	15a	Describe criteria under which study data will be quantitatively synthesised	Study populations must include youth team sport athletes (under 18 yrs old). The independent variables must include a measure of internal or external workload. The dependent variable must be a measure of injury prevalence, incidence, relative risk and/or odds ratio.
	15b	If data are appropriate for quantitative synthesis, describe planned summary measures, methods of handling data and methods of combining data from studies, including any planned exploration of consistency (such as I^2, Kendall's τ)	The following information will be extracted from each study<br>• Study design<br>• Population characteristics (age, sex, sport)<br>• Workload definition and measurement<br>• Injury definition and measurement<br>• Relative Risk and/or Odds Ratio measurements<br>Heterogeneity will be explored using I^2 statistic if possible. If considerable variation and inconsistency in study results exists, we will not pursue a meta-analysis.
	15c	Describe any proposed additional analyses (such as sensitivity or subgroup analyses, meta-regression)	If data quality and quantity allow, we will conduct subgroup analyses across different sports
	15d	If quantitative synthesis is not appropriate, describe the type of summary planned	If data quality and quantity are not sufficient for meta-analysis, a summary table with the extracted information will be presented.
Meta-bias(es)	16	Specify any planned assessment of meta-bias(es) (such as publication bias across studies, selective reporting within studies)	Meta-biases will not be assessed in this review.
Confidence in cumulative evidence	17	Describe how the strength of the body of evidence will be assessed (such as GRADE)	If conclusions are able to be drawn from the body of evidence, we will assess quality of evidence using the Grading of Recommendations Assessment, Development and Evaluation process. Specifically, quality will be based on risk of bias.

*** It is strongly recommended that this checklist be read in conjunction with the PRISMA-P Explanation and Elaboration (cite when available) for important clarification on the items. Amendments to a review protocol should be tracked and dated. The copyright for PRISMA-P (including checklist) is held by the PRISMA-P Group and is distributed under a Creative Commons Attribution Licence 4.0.**

From: Shamseer L, Moher D, Clarke M, Ghersi D, Liberati A, Petticrew M, Shekelle P, Stewart L, PRISMA-P Group. Preferred reporting items for systematic review and meta-analysis protocols (PRISMA-P) 2015: elaboration and explanation. BMJ. 2015 Jan 2;349(jan02 1):g7647.
