# Supplementary material for: Is Cumulative Load Associated with Injuries in Youth Team Sport? A Systematic Review
Source: Sports Med Open. 2022 Sep 16;8:117. doi: 10.1186/s40798-022-00516-w (PMC9481825; doi:10.1186/s40798-022-00516-w)
Supplement: Supplementary file 3 — Additional file 3: Table S2. Modified Newcastle Ottawa scale for cohort studies. [file 40798_2022_516_MOESM3_ESM.pdf]

Supplementary Table 2. Modified Newcastle Ottawa Scale for Cohort Studies.

Criteria	Evaluation
1. Representativeness of the exposed cohort	a) Truly representative of the average (youth team sport athlete) in the community*<br>b) Somewhat representative of the average (youth team sport athlete) in the community*<br>c) Selected group of users (eg nurses, volunteer)<br>d) No description of the derivation of the cohort
2. Ascertainment of exposure	e) Secure record (eg surgical records)*<br>f) Structured interview<br>g) Written self-report*<br>h) No description
3. Demonstration that outcome of interest was not present at start of study	a) Yes*<br>b) No
4. Assessment of outcome	a) Independent blind assessment*<br>b) Record linkage*<br>c) Self-report<br>d) No description
5. Was follow-up long enough for outcomes to occur	a) Yes*<br>b) No
6. Adequacy of follow-up of cohorts	a) Complete follow-up – all subjects accounted for*<br>b) Subject lost to follow-up unlikely to introduce bias (> %, or no description of those lost)*<br>c) Follow-up rate < % and no description of those lost<br>d) No statement
7. Definition of injury	a) Presents a definition of an injury informing what was considered as an injury in the study*<br>b) No definition of injury

* Studies with this assessment received a star for this criteria, maximum of one star per criteria
