# Supplementary material for: Is Cumulative Load Associated with Injuries in Youth Team Sport? A Systematic Review
Source: Sports Med Open. 2022 Sep 16;8:117. doi: 10.1186/s40798-022-00516-w (PMC9481825; doi:10.1186/s40798-022-00516-w)
Supplement: Supplementary file 4 — Additional file 4: Table S3. Modified Newcastle Ottawa scale for cross-sectional studies. [file 40798_2022_516_MOESM4_ESM.pdf]

Supplementary Table 3. Modified Newcastle Ottawa Scale for Cross-sectional Studies.

Criteria	Evaluation
1. Is the case definition adequate?	a) Yes, with independent validation*<br>b) Yes, eg record linkage or based on self reports<br>c) No description
2. Representativeness of cases	a) Consecutive or obviously representative series of cases*<br>b) Potential for selection biases or not stated
3. Selection of controls	a) Community controls*<br>b) Hospital controls<br>c) No description
4. Definition of controls	a) No history of disease (endpoint)*<br>b) No description of source
5. Comparability of cases and controls on the basis of the design or analysis	a) Study controls for " "*<br>b) Study control for any additional factor*
6. Ascertainment of exposure	a) Secure record (eg surgical records)*<br>b) Structured interview where blind to case/control status*<br>c) Interview not blinded to case/control status<br>d) Written self report or medical record only<br>e) No description
7. Same method of ascertainment for cases and controls	a) Yes*<br>b) No
8. Non-response rate	a) Same rate for both groups*<br>b) Non-respondents described<br>c) Rate different and no designation
9. Definition of injury	a) Presents a definition of an injury informing what was considered as an injury in the study*<br>b) No definition of injury

* Studies with this assessment received a star for this criteria
