# Supplementary material for: Is Cumulative Load Associated with Injuries in Youth Team Sport? A Systematic Review
Source: Sports Med Open. 2022 Sep 16;8:117. doi: 10.1186/s40798-022-00516-w (PMC9481825; doi:10.1186/s40798-022-00516-w)
Supplement: Supplementary file 5 — Additional file 5: Table S4. Modified Newcastle Ottawa scale scores. [file 40798_2022_516_MOESM5_ESM.pdf]

Supplementary Table 4. Modified Newcastle Ottawa Scale Scores

Reference	Reviewer 1	Reviewer 2	Tiebreaker	Final Score
Ahmun et al., 2019	6	5	6	6
Bowen et al., 2017	4	4	-	4
Brink et al., 2010	4	4	-	4
Frisch et al., 2017	3	3	-	4
Hartwig et al., 2019	3	3	-	3
Lathlean et al., 2020	7	5	5	5
O'Keeffe et al., 2020	6	6	-	6
Orr and Cheng, 2016	6	4	5	5
Ristolainen et al., 2019	7	5	7	7
Visnes and Barr, 2013	6	5	5	5
Watson et al., 2017	5	5	-	5
